# Leveraging hepatitis B vaccination to consolidate functional cure: reigniting the humoral axis in chronic HBV infection

**DOI:** 10.1080/22221751.2025.2547723

**Published:** 2025-08-13

**Authors:** Shan Ren, Sujun Zheng, Xinyue Chen

**Affiliations:** First Department of Liver Disease Center, Beijing Youan Hospital, Capital Medical University, Beijing, People’s Republic of China

**Keywords:** Hepatitis B, functional cure, anti-HBs, Peg-IFNα, vaccination

Functional cure for chronic hepatitis B(CHB)represents the current optimal treatment endpoint. It is defined as sustained hepatitis B surface antigen (HBsAg) loss (with undetectable serum HBV DNA and hepatitis B e antigen [HBeAg]) maintained for at least 24 weeks after treatment cessation. This endpoint is strongly associated with improved long-term clinical outcomes and a significantly reduced risk of hepatocellular carcinoma [1–4]. Functional cure can be achieved through pegylated interferon alpha (Peg-IFNα), nucleos(t)ide analogues (NAs), or spontaneous loss (with the latter two occurring less frequently [5]). However, HBsAg reversion rates range from 4.1% to 27.6% [6–10]. Notably, patients cured via Peg-IFNα exhibit the highest relapse risk (5-year reversion rate: 27.6% vs. 3.3% in NA-treated patients [7]), potentially linked to incomplete immune reconstitution after therapy withdrawal. Anti-HBs antibody level is a key determinant of durable Peg-IFNα induced functional cure. Patients achieving anti-HBs ≥ 100 mIU/mL at end-of-treatment (EOT) had significantly lower 1-year reversion rates than those with low/undetectable levels (5.5% vs. 21.7%−29.5%; *P* = 0.005 [10]). Our prospective study [9] further confirmed this quantitative relationship: durable cure rates were significantly higher in patients with EOT anti-HBs > 100 mIU/mL compared to those with ≤ 100 mIU/mL (95.7% vs. 78.9%; *P* < 0.001). Given the critical role of anti-HBs levels in sustaining Peg-IFNα induced functional cure, strategies to enhance these titres, particularly hepatitis B vaccination, are gaining attention as a focus of recent clinical research. Based on current evidence and clinical experience, we offer preliminary recommendations regarding these strategies as follows (Figure 1):

**Figure 1.**
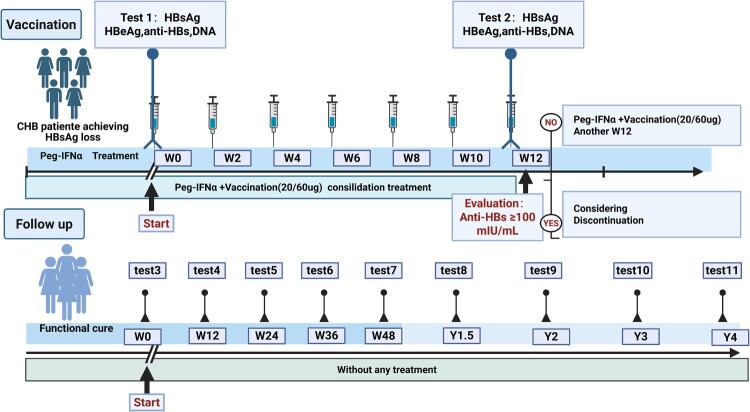
Clinical Implementation Strategy for therapeutic vaccination in chronic hepatitis B functional cure.

## Timing of vaccination

Initiate vaccination promptly post-HBsAg loss, concurrently with Peg-IFNα consolidation therapy. Standalone vaccination has shown limited efficacy, primarily due to persistent HBsAg maintaining immune tolerance via multifaceted immunosuppression (e.g. T/B/NK dysfunction,myeloid shifts) [11–14]; antigen reduction is prerequisite for immune reconstitution. Sustained HBsAg expression disrupts B-cell differentiation/function, hindering antibody production [15–18]. Sequential therapy (antigen reduction followed by vaccination) effectively enhances HBsAg loss.

(Flowchart summarizes the intervention protocol following HBsAg seroclearance) Initiation: Upon confirmed HBsAg loss, patients immediately receive 20 or 60 μg hepatitis B vaccine with concomitant pegylated interferon alpha (Peg-IFNα) therapy. Monitoring: Anti-HBs titres are quantified at 3 months. Patients achieving ≥100 mIU/mL discontinue treatment (functional cure endpoint). Extension: Suboptimal responders (<100 mIU/mL) extend combined therapy to 6 months. Surveillance: Post-treatment monitoring includes quarterly HBsAg and HBV DNA testing during Year 1, transitioning to biannual assessment thereafter. Follow-up continues through Year 5 to confirm sustained functional cure.

**Figure 2.**
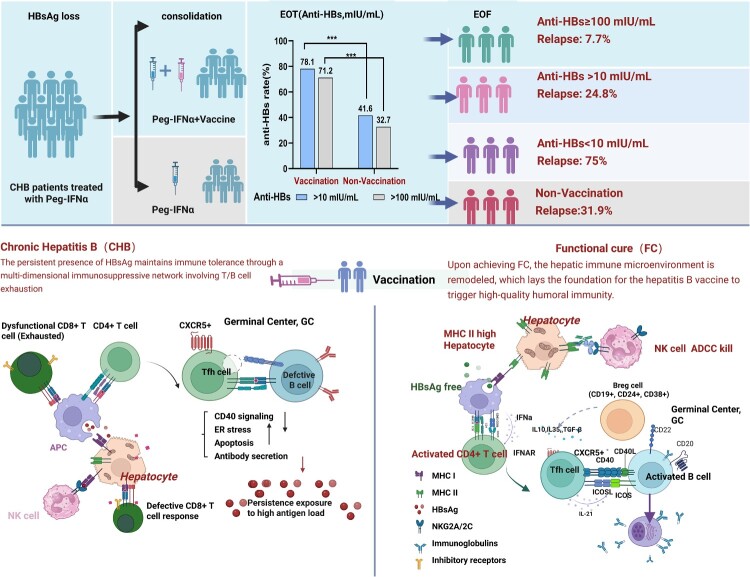
Triple Mechanisms Underlying Hepatitis B Vaccine Efficacy in Functional Cure(Integrated schematic of immunological remodelling and clinical outcomes)Upon hepatitis B surface antigen (HBsAg) seroclearance, the reconstituted hepatic microenvironment enables therapeutic vaccination to drive functional cure through three synergistic mechanisms. Panel A: B-cell Compartment Remodeling Illustrates HBsAg clearance-induced resolution of endoplasmic reticulum(ER)stress in B cells, enabling differentiation of memory B cells (CD19+/CD24+/CD38+) into antibody-secreting plasma cells within GCs. Panel B: Tfh Activation Depicts vaccine-induced expansion of ICOS^+^ CXCR5^+^ Tfh cells producing IL-21, which promotes B-cell maturation and anti-HBs antibody production. Tfh frequency correlates with anti-HBs titres (r = 0.62, *P* < 0.001; ref. 18). Panel C: Alleviation of Immune Suppression Demonstrates downregulation of inhibitory pathways post-vaccination, including reduction in regulatory B cells (Bregs) and CTLA-4^+^ exhausted T cells, thereby restoring CD8^+^ T-cell cytotoxicity. Inset: Clinical Correlate of Humoral Protection. Bar chart comparing clinical relapse rates by post-treatment anti-HBs levels: patients maintaining ≥100 mIU/mL exhibit significantly lower relapse (7.7%) versus those with <100 mIU/mL (58.5%; *P* < 0.001; refs. 7,24). Abbreviations: ADCC, antibody-dependent cellular cytotoxicity; EOT, end of treatment; FC, functional cure; GC, germinal centre; Tfh, T follicular helper.

Post-loss hepatic immune remodelling (e.g. reduced PD-1^+^CD8^+^ T cells/FOXP3^+^ Tregs, increased CD4^+^CTLs, elevated CCR7^low^PD-1^high^ Tfh correlating with anti-HBs/HBc) [19–22] may enable vaccine responsiveness,facilitating robust humoral immunity. Concurrent Peg-IFNα combined with vaccination [23] may synergize via: (1) promoting B cell differentiation into plasma cells, (2) activating ICOS^+^ Tfh for B-cell maturation, and (3) alleviating Breg/CTLA-4 suppression. This provides an immunological basis for rapid anti-HBs generation and reduced relapse risk (Figure 2).

## Optimizing dosing regimens

Based on accumulating evidence [7,23,24], an intensified regimen of 20 or 60 μg recombinant hepatitis B vaccine administered biweekly (over 3–6 months) is proposed. The primary objective is to induce anti-HBs levels ≥ 100 mIU/mL, a threshold associated with reduced HBsAg reversion risk and durable functional cure.

Central to this paradigm is the ≥100 mIU/mL anti-HBs threshold for sustained remission. A retrospective study [7] of 108 CHB patients achieving HBsAg loss with Peg-IFNα (72 received standard 0-1-6-month vaccination) demonstrated: Non responders (<10 mIU/mL)had significantly higher 5-year cumulative reversion than responders > 10 mIU/mL (75.0% vs 24.8%;log-rank *P* < 0.001), confirming extreme relapse risk in immunological non-responders; Responders > 10mIU/mL showed comparable reversion to unvaccinated controls (24.8% vs 31.9%; *P* = 0.459), indicating insufficient protection at >10 mIU/mL;Among responders, strong responders (≥100 mIU/mL) had only 7.7% reversion, establishing ≥100 mIU/mL as the critical threshold for effective immune reconstitution. Conversely, weak responders (10-99 mIU/mL) exhibited higher reversion than unvaccinated controls (58.5% vs 31.9%; *P* = 0.039), suggesting suboptimal antibody levels may permit residual viral activity (e.g. cccDNA reactivation) and paradoxically increase relapse risk. This apparent contradiction (comparable reversion in overall responders vs controls) does not negate vaccination value, but underscores the absolute necessity of sufficiently high antibody levels to reduce relapse in Peg-IFNα-induced HBsAg loss patients.

Building on this rationale, intensified vaccination schedules (biweekly for ≥3months) demonstrate superior efficacy. Our study [24] confirmed that during Peg-IFNα consolidation, biweekly 20 μg vaccination for ≥3 months significantly increased: Anti-HBs seroconversion (78.1% vs 41.6%); ≥ 100 mIU/mL (71.2% vs 32.7%) and ≥300 mIU/mL rates (56.2% vs 17.8%) (all *P* < 0.001) without additional safety risks. Based on the confirmed efficacy of intensified vaccination frequency in enhancing response levels, further optimization of vaccine dosage emerges as a critical strategy to potentially augment immunoprotective efficacy. While current studies predominantly utilize the 20μg vaccine, consideration of the 60μg high-dose formulation, where accessible, is warranted given its superior immunogenicity profile, with the expectation that higher dosing may further optimize antibody responses. In healthy adults [25,26], switching 20μg non-responders to a single 60μg dose significantly improved anti-HBs seroconversion rates to 91.7%, compared to an 87.1% rate achieved with multiple 20μg doses. In immunocompromised populations (hemodialysis patients and HIV-infected individuals) [27,28], a standard 0-1-6 month schedule using the 60μg vaccine induced significantly higher antibody seropositivity rates and geometric mean titres (GMT) than the 20μg vaccine (*P* < 0.05), and demonstrated superior 5-year immunogenicity persistence.

## Monitoring strategy and individualized adjustment

Current evidence identifies anti-HBs levels >100 mIU/mL as a key predictor for sustained remission after Peg-IFNα-induced functional cure (OR = 0.525,*P* < 0.001) [8,10]. Therefore, patients achieving HBsAg loss following 3 months of Peg-IFNα plus hepatitis B vaccine consolidation therapy with anti-HBs ≥ 100 mIU/mL may be considered for treatment cessation. Conversely, for those with persistent anti-HBs <100 mIU/mL, individualized decisions regarding extending therapy to 6 months are advised, based on a comprehensive assessment of baseline characteristics (e.g. prior treatment response patterns, cirrhosis status) and cumulative Peg-IFNα duration.

Systematic post-therapy monitoring is essential. Given relapse risk patterns, the regimen comprises: quarterly Year 1 assessments (M3,6,9,12) with HBV markers, HBV DNA, liver function (LFTs), and complete blood count (CBC) at all visits; AFP, ultrasound, and LSM additionally at M6/12. Biannual Year 2 (M18,24) and annual Years 3–5 assessments all include the full panel. Minimum follow-up is 5 years post-cessation. This monitoring strategy is supported by: (1) The observation that 21.9% of relapses manifest as HBV DNA positivity despite HBsAg negativity (Type III relapse) [29], underscoring the necessity of concurrent serological and virological monitoring; (2) Time-window analysis (median follow-up 160 weeks in 238 Peg-IFNα-induced HBsAg clearance patients) [7,9] revealing an overall relapse rate of 7.6% (18/238), with 72.2% (13/18) occurring within 52 weeks post-treatment; notably, patients with anti-HBs <100 mIU/mL relapsed significantly earlier than those with ≥100 mIU/mL (median 41 weeks vs. 107 weeks; *P* < 0.001) [7]; and (3) Long-term data indicating an approximate 10-year cumulative relapse rate of 10%, with >90% of relapses occurring within the first 5 years post-cessation [30], justifying the 5-year monitoring duration.

Phase II data for the novel multi-antigen therapeutic vaccine BRII-179 (containing PreS1/S2/HBsAg), presented at the 2023 AASLD meeting, demonstrated that Peg-IFNα combined with BRII-179 significantly increased HBsAg seroconversion rates compared to Peg-IFNα with placebo (15.8% vs. 1.8%, *P* < 0.05) in 114 NA-experienced CHB patients [31]. Further mechanistic studies revealed that combining BRII-179 with siRNA (BRII-835) synergistically enhanced both anti-HBs levels and HBsAg-specific T-cell responses, inducing a Th1-polarized cytokine signature particularly in patients achieving substantial HBsAg decline [32]. Critically, Th1 immunity is essential for effective antiviral defense. The persistence of HBV-specific T-cell responses and Th1 signatures observed in patients maintaining functional cure suggests the establishment of immunological memory. Collectively, these findings underscore that sustained functional cure relies on coordinated B- and T-cell immune reconstitution. Analogous to how virus-specific antibodies signify protective immunity following hepatitis A virus (HAV) or other acute viral infections, the concerted activation of B-cell (humoral) and T-cell (cellular) immunity is fundamental for achieving durable virological control in HBV infection.
